# Automated Endocardial Border Detection and Left Ventricular Functional Assessment in Echocardiography Using Deep Learning

**DOI:** 10.3390/biomedicines10051082

**Published:** 2022-05-06

**Authors:** Shunzaburo Ono, Masaaki Komatsu, Akira Sakai, Hideki Arima, Mie Ochida, Rina Aoyama, Suguru Yasutomi, Ken Asada, Syuzo Kaneko, Tetsuo Sasano, Ryuji Hamamoto

**Affiliations:** 1 Department of Cardiovascular Medicine, Tokyo Medical and Dental University, 1-5-45 Yushima, Bunkyo-ku, Tokyo 113-8510, Japan; 180181ms@tmd.ac.jp (S.O.); hideki-arima.cvm@tmd.ac.jp (H.A.); ochimlab@tmd.ac.jp (M.O.); sasano.cvm@tmd.ac.jp (T.S.); 2 Division of Medical AI Research and Development, National Cancer Center Research Institute, 5-1-1 Tsukiji, Chuo-ku, Tokyo 104-0045, Japan; raizawa@ncc.go.jp (R.A.); ken.asada@riken.jp (K.A.); sykaneko@ncc.go.jp (S.K.); 3 Cancer Translational Research Team, RIKEN Center for Advanced Intelligence Project, 1-4-1 Nihonbashi, Chuo-ku, Tokyo 103-0027, Japan; 4 Artificial Intelligence Laboratory, Research Unit, Fujitsu Research, Fujitsu Ltd., 4-1-1 Kamikodanaka, Nakahara-ku, Kawasaki 211-8588, Japan; akira.sakai@fujitsu.com (A.S.); yasutomi.suguru@fujitsu.com (S.Y.); 5 RIKEN AIP-Fujitsu Collaboration Center, RIKEN Center for Advanced Intelligence Project, 1-4-1 Nihonbashi, Chuo-ku, Tokyo 103-0027, Japan; 6 Department of NCC Cancer Science, Biomedical Science and Engineering Track, Graduate School of Medical and Dental Sciences, Tokyo Medical and Dental University, 1-5-45 Yushima, Bunkyo-ku, Tokyo 113-8510, Japan

**Keywords:** deep learning, echocardiography, endocardial border detection, left ventricular ejection fraction, myocardial strain assessment

## Abstract

Endocardial border detection is a key step in assessing left ventricular systolic function in echocardiography. However, this process is still not sufficiently accurate, and manual retracing is often required, causing time-consuming and intra-/inter-observer variability in clinical practice. To address these clinical issues, more accurate and normalized automatic endocardial border detection would be valuable. Here, we develop a deep learning-based method for automated endocardial border detection and left ventricular functional assessment in two-dimensional echocardiographic videos. First, segmentation of the left ventricular cavity was performed in the six representative projections for a cardiac cycle. We employed four segmentation methods: U-Net, UNet++, UNet3+, and Deep Residual U-Net. UNet++ and UNet3+ showed a sufficiently high performance in the mean value of intersection over union and Dice coefficient. The accuracy of the four segmentation methods was then evaluated by calculating the mean value for the estimation error of the echocardiographic indexes. UNet++ was superior to the other segmentation methods, with the acceptable mean estimation error of the left ventricular ejection fraction of 10.8%, global longitudinal strain of 8.5%, and global circumferential strain of 5.8%, respectively. Our method using UNet++ demonstrated the best performance. This method may potentially support examiners and improve the workflow in echocardiography.

## 1. Introduction

Two-dimensional (2D) echocardiography is extensively utilized in cardiovascular examination owing to its real-time and non-invasive nature. This imaging modality allows us to assess not only cardiovascular morphology but also its function with several quantitative or qualitative dynamic analyses, including Doppler imaging and regional wall motion analysis. However, echocardiographic images are acquired through manual sweep scanning, which means that the image quality and diagnostic accuracy depend on the skill levels of the examiners. Echocardiographic technologies, such as three-dimensional (3D) echocardiography and myocardial deformation imaging, have gradually evolved to increase the ability of the scanning probe, image quality, or accuracy of functional analyses [1]. However, these latest technologies still demand expertise for acquiring images with acceptable quality in 2D echocardiography.

Concerning echocardiographic functional analysis, the assessment of left ventricular systolic function is fundamental in diagnosing and managing cardiovascular diseases. The left ventricular ejection fraction (LVEF) is one of the major established echocardiographic indexes. LVEF is calculated from the end-diastolic volume and the end-systolic volume estimates by the biplane disk summation method (modified Simpson’s rule) based on left ventricular endocardial border detection [2]. Myocardial strain assessment has also developed along with the current technological progress in myocardial deformation imaging. Because of the incidence of several types of heart failure that exhibit preserved ejection fraction, myocardial strain assessment is expected to be useful for the early detection of the symptoms of these cardiovascular diseases. Strains can be analyzed in three directions: longitudinal, circumferential, and radial. Among these strain indexes, the highest level of clinical evidence has been accumulated for the global longitudinal strain (GLS) [3]. Prior studies have reported that the reproducibility of GLS is superior to LVEF [4]. GLS defines the relative change of the left ventricular myocardial length between end-diastole and end-systole [5]. This index is usually derived from the peak value of 2D longitudinal speckle tracking, which also requires endocardial border detection [6].

As mentioned above, endocardial border detection is a key step in assessing left ventricular systolic function. Currently, several commercially available ultrasound machines are equipped with semi-automatic techniques to detect the endocardial border [7]. However, their endocardial border detection lacks accuracy, meaning that examiners often have to fix the initial endocardial contour manually in clinical practice. This subjective process is time-consuming and causes differences among examiners and devices. Therefore, further accurate and normalized automatic endocardial border detection would be valuable.

Artificial intelligence (AI), including machine learning and deep learning, has developed remarkably and has since been applied to a wide range of medical research topics [8,9,10,11,12,13]. AI has the potential to achieve tasks more rapidly and accurately than humans, especially in the field of medical imaging [14,15,16]. However, data acquisition with manual sweep scanning and acoustic shadows makes AI-based ultrasound imaging analysis more difficult than other medical imaging modalities. This deterioration in practical performance needs to be addressed by utilizing specialized algorithms and preprocessing [17,18,19]. The clinical applications of AI may support examiners and improve the workflow in ultrasound imaging [20]. Prominent efforts have been made in medical AI research of echocardiography. Reportedly, the automated machine learning algorithm could be used to quickly measure dynamic left ventricular and atrial volumes in 3D echocardiography [21]. A method to detect cardiac events in echocardiography using 3D convolutional recurrent neural networks was developed [22]. Salte et al. proposed a fully automated pipeline to measure GLS using a motion estimation technology based on deep learning [23].

In this study, we introduce state-of-the-art segmentation methods of the left ventricular cavity in six representative projections in 2D echocardiographic videos. We compare their performance in endocardial border detection and left ventricular functional assessment.

## 2. Materials and Methods

### 2.1. Data Preparation

A total of 3938 ultrasound images from 154 echocardiographic videos of 29 subjects were used in this study. All subjects underwent echocardiography after providing written informed consent at the Tokyo Medical and Dental University Hospital (Tokyo, Japan) according to the guidelines of the American Society of Echocardiography (ASE) and the European Association of Cardiovascular Imaging (EACVI) [2]. All participants were men, and their mean age was 37 (20–60). The cohort comprised 27 healthy volunteers and 2 patients with cardiac diseases (bicuspid aortic valve and hypertensive heart disease). All subjects were enrolled in research protocols approved by the Institutional Review Board of RIKEN, Fujitsu Ltd., Tokyo Medical and Dental University, and the National Cancer Center Japan (approval ID: Wako3 2019-36). Echocardiographic videos were acquired by board-certified specialists in echocardiography using Vivid E95^®^ (GE Healthcare, Chicago, IL, USA) or EPIQ CVx^®^ (Philips Healthcare, Amsterdam, The Netherlands). There was no bias in image quality caused by the examiner acquiring the echocardiographic videos. The dataset of each subject involved several videos of the six representative projections, including apical two-chamber view (2CV), apical three-chamber view (3CV), apical four-chamber view (4CV), parasternal short-axis views at the apex (SA), mitral valve (SM), and papillary muscle level (SP). All methods were performed in accordance with the Ethical Guidelines for Medical and Health Research Involving Human Subjects. With regard to the handling of data, we followed the Data Handling Guidelines for the Medical AI Project at the National Cancer Center Japan (ver.3.6 (2021)).

### 2.2. Data Preprocessing and Augmentation

The actual sections of the left ventricular endocardium were annotated pixel-by-pixel under the supervision of two cardiologists specializing in echocardiography to create the correct answer labels. A dataset of 23 healthy volunteers for training and a dataset of 4 residual healthy volunteers and 2 patients for the test data were randomly employed. The training dataset included 2798 images from 118 videos, and the training images of each projection were assigned a ratio of 4:1, which corresponded to a ratio of the training to validation data. None of the subjects straddled the training and validation datasets. The test dataset comprised 1140 images from 36 videos, which equally consisted of 6 videos per projection (Table S1).

Since the amount of our data was limited, data augmentation was performed. Rotation, brightness, and contrast were changed for the training data (Figure S1). The image was rotated in the range of ±15 degrees. For brightness and contrast, the following conversion was performed using function src as the original image and function dst as the output image:

(1) $$\mathrm{dst}\left(I\right)=\mathrm{saturate}\_\mathrm{cast}\left(\left|\mathrm{src}\left(I\right)\times \alpha +\beta \right|\right).$$

The *α* ranged from 0.7 to 1.3 and *β* from −30 to 30. As a result of the data augmentation, the training data for each projection increased 21-fold.

### 2.3. Endocardial Border Detection and Left Ventricular Functional Assessment

**Figure 1:**
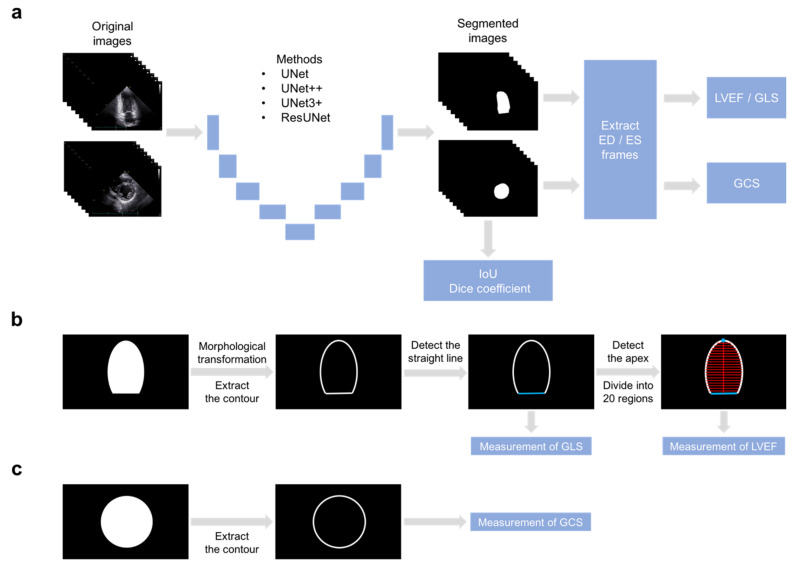
Flow chart of endocardial border detection and left ventricular functional assessment. (**a**) Four segmentation methods of the left ventricular cavity were evaluated in the six projections. After automatic detection of end-diastolic and end-systolic frames and extraction of the contour as an endocardial border, the echocardiographic indexes were measured using the apical chamber views (**b**) and the parasternal short-axis views (**c**). ED, end-diastolic; ES, end-systolic; LVEF, left ventricular ejection fraction; GLS, global longitudinal strain; GCS, global circumferential strain.

Figure 1 shows a flow chart of the method used for endocardial border detection and left ventricular functional assessment. First, segmentation of the left ventricular cavity was performed in the six representative projections for a cardiac cycle. We employed four segmentation methods: U-Net [24], UNet++ [25], UNet3+ [26], and Deep Residual U-Net (ResUNet) [27]. The input and output images were resized to 256 × 256 pixels. Hyperparameters for each method were retrieved from the literature.

Next, an end-diastolic frame and an end-systolic frame were detected from each echocardiographic video to measure LVEF and myocardial strain. According to the guidelines [2], end-diastole is preferably defined as the first frame after mitral valve closure or the frame in the cardiac cycle in which the respective left ventricular dimension or volume measurement is the largest. End-systole is best defined as the frame after aortic valve closure or the frame in which the cardiac dimension or volume is smallest. In this study, we defined the peak of the QRS complex as end-diastole. Therefore, an end-diastolic frame could be detected with the highest point of a red marker in the electrocardiogram on the echocardiographic video using Vivid E95^®^. Because aortic valve closure could not be detected in this study, end-systole was defined as when the left ventricular segmentation area was minimum in a single cardiac cycle. Thus, we developed an automatic detection method for end-diastolic and end-systolic frames (Figure S2).

Additionally, the mitral valve annulus was detected to measure LVEF and GLS using the apical chamber views. Since the contour of the segmentation image is uneven, the contour was smoothed by morphology processing. Subsequently, we detected a straight line on the contour by Hough transform and both endpoints of the straight line as mitral valve annulus. Furthermore, the apex of the heart was detected with the largest Euclidean distance between the contour of the segmentation image and the midpoint of the mitral valve annulus to measure LVEF (Figure 1b). Regarding the measurement of global circumferential strain (GCS) using parasternal short-axis views, we extracted the contour of the segmentation image at the end-diastolic and end-systolic frames (Figure 1c).

### 2.4. Metrics

#### 2.4.1. Segmentation Performance

Intersection over Union (IoU) and the Dice coefficient (Dice) are generally used to quantify the performance of segmentation methods. When true-positive pixels are defined as TP, false-positive pixels as FP, and false-negative pixels as FN, these indexes are calculated as follows:

(2) $$\mathrm{IoU}=\frac{\mathrm{TP}}{\mathrm{TP}+\mathrm{FP}+\mathrm{FN}}\;\mathrm{Dice}=\frac{2\mathrm{TP}}{2\mathrm{TP}+\mathrm{FP}+\mathrm{FN}}.$$

These metrics take values between 0 and 1, with values closer to 1 corresponding to better predictions. For each of the four segmentation methods, both IoU and Dice were calculated for all frames of each projection. For the inference results and correct labels, the mean value of IoU (mIoU), the mean value of Dice (mDice), and the standard deviation were calculated for each projection. The performance of four segmentation methods was evaluated using mIoU and mDice.

#### 2.4.2. LVEF

The biplane disk summation method (modified Simpson’s rule) is currently recommended to assess LVEF by consensus of the committee of ASE and EACVI [2]. According to this method, we divided the long axis (*L*) of the apical two-chamber view and the apical four-chamber view into 20 disks, determined the inner diameter (*a_i_*, *b_i_*) of the short axis orthogonal to the long axis, and then assumed the volume of each disk as an elliptical column. The volume (*V*) was calculated using the following formula:

(3) $$V=\frac{\pi }{4}\overset{20}{\underset{i=1}{\sum }}a_{i}b_{i}\frac{L}{20}.$$

LVEF is defined as the ratio of left ventricular stroke volume to left ventricular end-diastolic volume. The stroke volume of the left ventricle was calculated by subtracting the end-systolic volume (ESV) from the end-diastolic volume (EDV). Therefore, LVEF was calculated as follows:

(4) $$\mathrm{LVEF}\;\left[\%\right]=\frac{\mathrm{EDV}-\mathrm{ESV}}{\mathrm{EDV}}\times 100.$$

#### 2.4.3. GLS and GCS

Myocardial strain assessment is used to evaluate the left ventricular systolic function that cannot be stratified by LVEF. Regarding GLS, clinical evidence has accumulated, and it is expected to be useful for the early detection of heart failure with preserved ejection fraction and myocardial disorders related to anticancer drug treatment [3]. Based on the Lagrangian analysis, the global strain was defined as the relative shortening of the whole endocardial contour length [5]. Both GLS and GCS define the relative change of the endocardial border length of the left ventricle between end-systole (*L*_ES_) and end-diastole (*L*_ED_). GLS and GCS are calculated as follows:

(5) $$\mathrm{GLS}\;\left[\%\right]=\frac{L_{\mathrm{ES}}-L_{\mathrm{ED}}}{L_{\mathrm{ED}}}\times 100\;\mathrm{GCS}\;\left[\%\right]=\frac{L_{\mathrm{ES}}-L_{\mathrm{ED}}}{L_{\mathrm{ED}}}\times 100.$$

According to the guidelines, GLS measurements should be made in the three standard apical views and averaged [2]. We further performed GCS measurements in the three standard parasternal short-axis views and calculated the average.

Estimation errors of LVEF, GLS, and GCS were evaluated between the correct value from the ground truth label and the estimated value using the segmentation image by each method. Since relative error can take both positive and negative values, we averaged the absolute values of the relative error. The accuracy of the four segmentation methods was evaluated by calculating the mean and median values for the absolute error of each index.

## 3. Results

### 3.1. Performance Comparison of the Segmentation Methods

**Figure 2:**
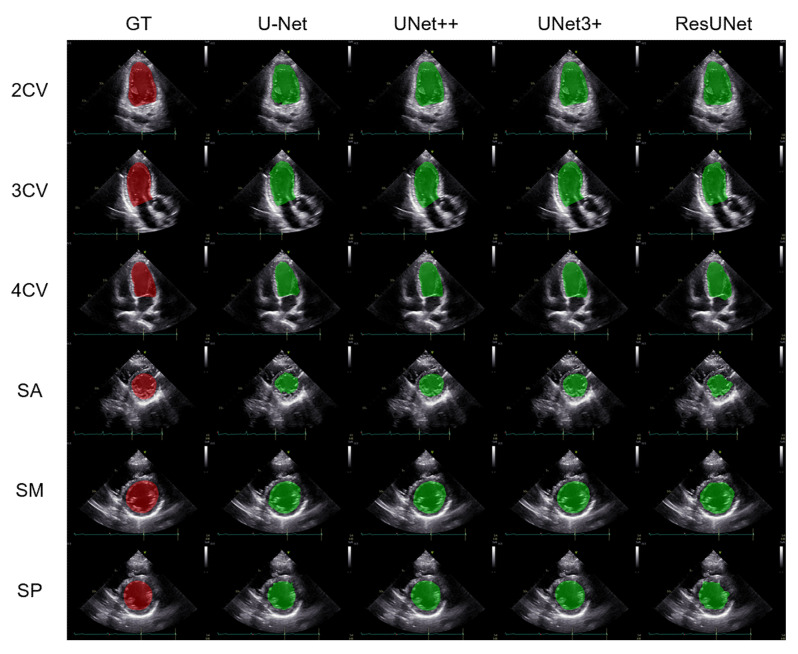
Representative segmentation images of the left ventricular cavity in the six projections for the 4 methods. The red region represents the ground-truth label, and the green region represents the segmented left ventricular cavity. GT, ground truth; 2CV, apical two-chamber view; 3CV, apical three-chamber view; 4CV, apical four-chamber view; SA, parasternal short-axis view (apex level); SM, parasternal short-axis view (mitral valve level); SP, parasternal short-axis view (papillary muscle level).

Figure 2 shows representative segmentation images of the left ventricular cavity in the six projections for U-Net, UNet++, UNet3+, and ResUNet, respectively. The upper three rows represent the apical chamber views, including 2CV, 3CV, and 4CV. The lower three rows represent the parasternal short-axis views, including SA, SM, and SP. The red region represents the ground-truth label, and the green region represents the segmented left ventricular cavity.

Table 1 shows the quantitative evaluation of segmentation results in the six projections for each method using mIoU and mDice. UNet++ yielded the highest values in 4CV, SM, and SP; the mIoU/mDice values were 0.871/0.929, 0.887/0.939, and 0.888/0.939, respectively. In contrast, UNet3+ yielded the highest values in 2CV, 3CV, and SA; mIoU/mDice were 0.891/0.942, 0.901/0.948, and 0.817/0.893, respectively. UNet++ and UNet3+ tended to demonstrate higher performance than U-Net and ResUNet in the experiment part of segmentation of the left ventricular cavity.

### 3.2. Left Ventricular Functional Assessment

**Figure 3:**
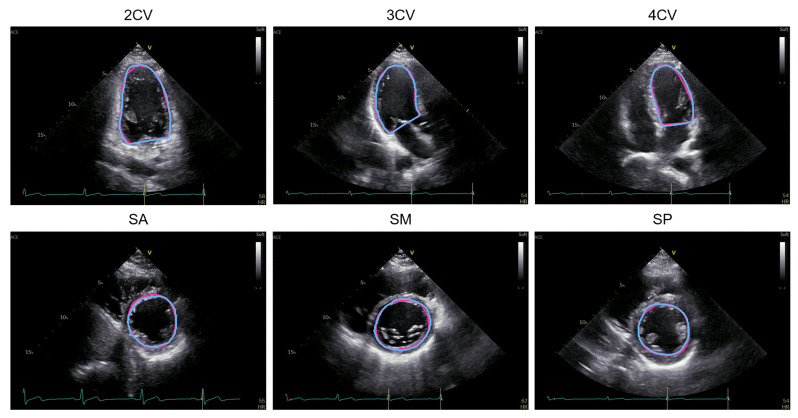
Representative estimated images of the endocardial border in the six projections using UNet++. The red line represents the ground-truth label, and the blue line represents the estimated endocardial border.

Based on the segmentation images of the left ventricular cavity at end-diastole and end-systole by each method, the contour of each segmented area was extracted as an endocardial border, and left ventricular functional assessment was conducted by measuring LVEF, GLS, and GCS. The representative estimated images and video of the endocardial border in the six projections using UNet++ are shown in Figure 3 and Video S1. The red line represents the ground-truth label, and the blue line represents the estimated endocardial border. The test dataset comprised 1140 images from 36 videos of 6 cases, which equally consisted of 6 videos per projection. The accuracy of the four segmentation methods was evaluated by calculating the mean and median values for the estimation error of each index in the test data (Table 2). The estimation error due to UNet++ was the smallest for LVEF, GLS, and GCS; the mean (median) values for the error were 10.8 (7.8)%, 8.5 (8.7)%, and 5.8 (5.2)%, respectively.

## 4. Discussion

To our knowledge, various AI-based analysis methods of ultrasound imaging have been previously reported, and the Food and Drug Administration in the United States has approved several AI-powered medical devices for ultrasound imaging [20]. However, difficulties and limitations in image quality control and acoustic shadows have affected and slowed the progress of medical AI research, as well as the development of ultrasound imaging compared to other medical imaging modalities. To address these characteristic problems of ultrasound imaging, we previously proposed segmentation methods using time-series information in ultrasound video [17,18] and the shadow estimation method using auto-encoder and synthetic shadows [19]. Furthermore, the clinical application of AI-powered medical devices remains challenging because of the black box problem; therefore, explainable AI needs to be considered in ultrasound imaging [28,29].

In this study, we focused on endocardial border detection and left ventricular functional assessment based on state-of-the-art segmentation methods of the left ventricular cavity in the six representative projections in 2D echocardiographic videos. As mentioned above, endocardial border detection is an important process, and several ultrasound machines are equipped with semi-automatic techniques to detect the endocardial border. However, these methods are still not sufficiently accurate, and manual retracing is often required and causes time-consuming and intra-/inter-observer variability in clinical practice. Moreover, there is a statistically significant variation in GLS measurement among vendors [7]. To address these clinical issues, developing accurate and normalized automatic endocardial border detection methods is important. Zyuzin et al. used U-Net to segment the left ventricular cavity in 4CV and identify the endocardial border on 2D echocardiographic images [30]. Their obtained accuracy (mDice) of left ventricular segmentation was 0.923. EchoNet-Dynamic, a video-based deep learning algorithm, segmented the left ventricle in 4CV on 2D echocardiographic videos with an mDice of 0.920 [31]. Wu et al. evaluated their semi-supervised model on two public echocardiographic video datasets, where mDice on the left ventricular endocardium segmentation achieved 0.929 and 0.938, respectively [32]. These reports demonstrated high segmentation performance of the left ventricular cavity, mainly in 4CV.

According to the ASE and EACVI guidelines, LVEF measurement by the biplane disk summation method (modified Simpson’s rule) is recommended, with reference to 2CV and 4CV. Furthermore, GLS measurements should be made in 2CV, 3CV, and 4CV and then averaged [2]. Although the clinical evidence of GCS remains limited, the clinical value of GCS could increase from now on. Therefore, segmentation of the left ventricular cavity was investigated in these six representative projections in this study. Regarding the segmentation of the left ventricle in the six projections, Kim et al. reported automatic segmentation of the left ventricle in echocardiographic images of pigs using convolutional neural networks. The mDice on the left ventricular cavity segmentation was 0.903 and 0.912 for U-Net and segAN, respectively [33]. We employed four segmentation methods: U-Net, UNet++, UNet3+, and ResUNet. In these analyses, UNet++ yielded the highest values in 4CV, SM, and SP, whereas UNet3+ yielded the highest values in 2CV, 3CV, and SA. Compared to the abovementioned research in terms of mDice, UNet++ and UNet3+ demonstrated sufficiently high performance in the experiment part of segmentation of the left ventricular cavity.

Subsequently, the accuracy of the four segmentation methods was evaluated by calculating the mean and median values for the estimation error of the echocardiographic indexes. Our result demonstrated that the estimation error due to UNet++ was the smallest for LVEF, GLS, and GCS. To assess these estimation errors, we should consider clinical intra-or inter-observer reproducibilities for these indexes. Chuang et al. reported an intra-observer score of 13.4% and inter-observer variability of 17.8% for LVEF in 2D echocardiography [34]. Referring to other reports, the inter-observer variation of LVEF can be as high as 13.9% [31,35]. Farsalinos et al. reported that intra-observer variability ranged from 4.9% to 7.3%, and inter-observer variability for GLS ranged from 5.3% to 8.6% [7]. In this study, UNet++ was superior to the other segmentation methods, with acceptable estimation accuracy of the echocardiographic indexes within clinical intra-/inter-observer variability. EchoNet-Dynamic predicted LVEF with the mean absolute error of 4.1% and 6.0% for two different datasets [31]. A prospective evaluation of the estimation accuracy of LVEF, GLS, and GCS using UNet++ for other datasets with repeated human measurements should be conducted in the future.

This study has several limitations. First, there was a limited number of test data from healthy volunteers and patients. To prove the clinical value of our method, we could have performed a prospective accuracy evaluation of our method using big datasets; we could have conducted a k-fold cross-validation and classified the subjects so as not to induce bias according to the clinical background, including age, sex, and types of cardiovascular diseases. Second, we did not evaluate the influence of acoustic shadows in 2D echocardiographic videos. Because acoustic shadows affect image quality control, shadow detection and other preprocessing may need to be considered in future studies. Finally, all data were acquired by board-certified specialists in echocardiography using the same type of ultrasound equipment; we did not experiment with examiners of all experience levels or with other equipment. These are important because statistically significant differences in image quality and echocardiographic index measurements can occur among examiners and vendors. The generalization of our method to examiners of all experience levels and equipment in a clinical scenario is a subject for future studies.

## 5. Conclusions

We developed a deep learning-based method for automated endocardial border detection and left ventricular functional assessment in 2D echocardiographic videos. Our method using Unet++ demonstrated the best performance and has the potential to support examiners and improve the workflow in echocardiography. For future work, to improve the accuracy of our method for clinical application, we should continue to acquire further echocardiographic videos and perform a prospective evaluation using big datasets. From another perspective, it may be necessary to develop an image quality evaluation technique that determines in advance whether the acquired echocardiographic video is a suitable input video for our method.

## Figures and Tables

**Table 1:** Evaluation of segmentation results in the six projections for each method using mIoU and mDice.

Method	Projection	mIoU	mDice
U-Net	2CV	0.855 ± 0.068	0.920 ± 0.041
	3CV	0.752 ± 0.137	0.851 ± 0.097
	4CV	0.816 ± 0.100	0.895 ± 0.063
	SA	0.670 ± 0.153	0.791 ± 0.125
	SM	0.841 ± 0.090	0.911 ± 0.057
	SP	0.813 ± 0.093	0.893 ± 0.062
UNet++	2CV	0.890 ± 0.042	0.941 ± 0.024
	3CV	0.886 ± 0.034	0.939 ± 0.019
	4CV	0.871 ± 0.067	0.929 ± 0.040
	SA	0.808 ± 0.125	0.887 ± 0.099
	SM	0.887 ± 0.066	0.939 ± 0.039
	SP	0.888 ± 0.064	0.939 ± 0.040
UNet3+	2CV	0.891 ± 0.039	0.942 ± 0.022
	3CV	0.901 ± 0.028	0.948 ± 0.016
	4CV	0.864 ± 0.063	0.926 ± 0.039
	SA	0.817 ± 0.116	0.893 ± 0.095
	SM	0.887 ± 0.079	0.938 ± 0.047
	SP	0.873 ± 0.084	0.930 ± 0.056
ResUNet	2CV	0.851 ± 0.056	0.919 ± 0.034
	3CV	0.837 ± 0.063	0.910 ± 0.038
	4CV	0.822 ± 0.088	0.900 ± 0.057
	SA	0.732 ± 0.155	0.834 ± 0.130
	SM	0.834 ± 0.090	0.907 ± 0.057
	SP	0.814 ± 0.082	0.895 ± 0.056

The values are mean ± standard deviation. mIoU, the mean value of Intersection over Union; mDice, the mean value of Dice.

**Table 2:** Accuracy evaluation of echocardiographic indexes for each method using the mean and median values for the estimation error.

Method	LVEF		GLS		GCS	
	Mean	Median	Mean	Median	Mean	Median
U-Net	24.3	23.3	36.4	37.7	17.7	14.7
UNet++	10.8	7.8	8.5	8.7	5.8	5.2
UNet3+	11.7	10.7	14.6	16.0	6.4	5.2
ResUNet	12.5	13.9	13.0	15.7	16.2	22.3

The values are estimation errors [%].

## Data Availability

Not applicable.

## Supplementary Materials

The following are available online at https://www.mdpi.com/article/10.3390/biomedicines10051082/s1, Figure S1: data augmentation, Figure S2: extraction of the end-diastolic frame and end-systolic frame, Table S1: data preparation for deep learning, Video S1: endocardial border detection in the six projections using Unet++.
