# Supplementary material for: Automated Endocardial Border Detection and Left Ventricular Functional Assessment in Echocardiography Using Deep Learning
Source: Biomedicines. 2022 May 6;10(5):1082. doi: 10.3390/biomedicines10051082 (PMC9138644; doi:10.3390/biomedicines10051082)
Supplement: Supplementary file 1 [file biomedicines-10-01082-s001.zip › Supplementary Table S1.pdf]

Supplementary Table S1

Projection	Train		Test	
	Movie	Image	Movie	Image
2CV	17	489	6	193
3CV	16	446	6	170
4CV	22	465	6	186
SA	20	385	6	170
SM	22	505	6	179
SP	21	508	6	242
Total	118	2798	36	1140

2CV, apical 2 chamber; 3CV, apical 3 chamber; 4CV, apical 4 chamber; SA, parasternal short axis (apex); SM, parasternal short axis (mitral valve); SP, parasternal short axis (papillary muscle).
