# Supplementary figures and images for: Automated Endocardial Border Detection and Left Ventricular Functional Assessment in Echocardiography Using Deep Learning
Source: Biomedicines. 2022 May 6;10(5):1082. doi: 10.3390/biomedicines10051082 (PMC9138644; doi:10.3390/biomedicines10051082)

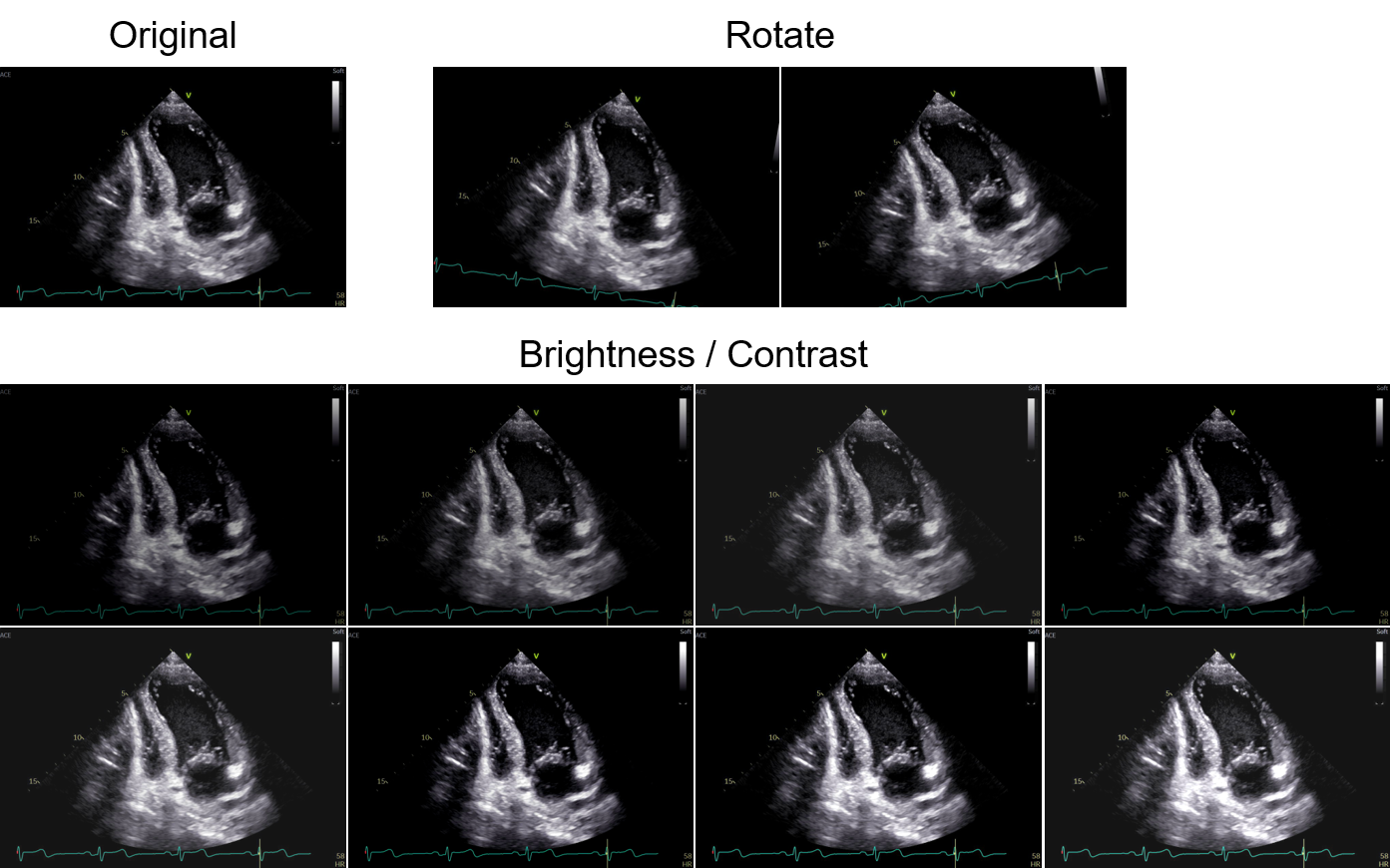

Supplement: Supplementary file 1 [file biomedicines-10-01082-s001.zip › Supplementary Figure S1.png]

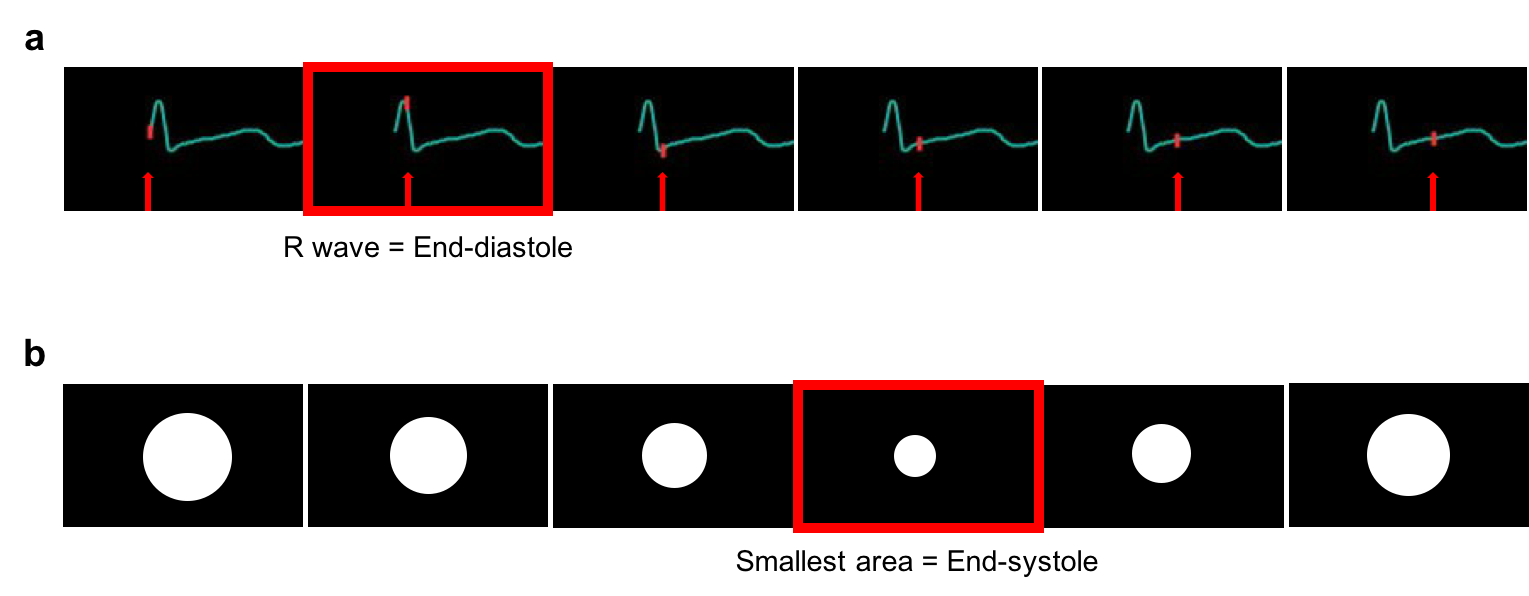

Supplement: Supplementary file 1 [file biomedicines-10-01082-s001.zip › Supplementary Figure S2.png]
